# Neurotensin Branched Peptide as a Tumor-Targeting Agent for Human Bladder Cancer

**DOI:** 10.1155/2015/173507

**Published:** 2015-04-23

**Authors:** Jlenia Brunetti, Chiara Falciani, Barbara Lelli, Andrea Minervini, Niccolò Ravenni, Lorenzo Depau, Giampaolo Siena, Eleonora Tenori, Stefano Menichetti, Alessandro Pini, Marco Carini, Luisa Bracci

**Affiliations:** ^1^Department of Medical Biotechnologies, University of Siena, 53100 Siena, Italy; ^2^Department of Urology, University of Florence, Careggi Hospital, 50139 Florence, Italy; ^3^Department of Chemistry, University of Florence, 50019 Florence, Italy

## Abstract

Despite recent advances in multimodal therapy, bladder cancer still ranks ninth in worldwide cancer incidence. New molecules which might improve early diagnosis and therapeutic efficiency for tumors of such high epidemiological impact therefore have very high priority. In the present study, the tetrabranched neurotensin peptide NT4 was conjugated with functional units for cancer-cell imaging or therapy and was tested on bladder cancer cell lines and specimens from bladder cancer surgical resections, in order to evaluate its potential for targeted personalized therapy of bladder cancer. Fluorophore-conjugated NT4 distinguished healthy and cancer tissues with good statistical significance (*P* < 0.05). NT4 conjugated to methotrexate or gemcitabine was cytotoxic for human bladder cancer cell lines at micromolar concentrations. Their selectivity for bladder cancer tissue and capacity to carry tracers or drugs make NT4 peptides candidate tumor targeting agents for tracing cancer cells and for personalized therapy of human bladder cancer.

## 1. Introduction

Specific targeting of tumor-associated antigens, selectively expressed or overexpressed by tumor cells, are the goal of modern cancer therapy aimed at increasing drug efficiency and decreasing its nonspecific toxicity. To achieve selective tumor treatment, diagnosis should provide information about expression of tumor-specific antigens that might be targeted by specific drugs or drug-carriers.

The use of peptides as tumor-targeting agents was envisaged years ago when it was found that receptors for different endogenous regulatory peptides are overexpressed in several primary and metastatic human tumors and can be used as tumor antigens [1, 2].

The bottleneck for development of peptides as drugs has always been their extremely short half-life, due to physiological degradation by peptidases and proteases. Different chemical modifications, which can be introduced to obtain stabilized analogues, may profoundly change peptide affinity or specificity. Coupling of peptides to effector units for tumor imaging or therapy may also interfere with peptide biological activity.

Peptides synthesized in an oligobranched form [3] retain peptide biological activity or even increase it through multivalent binding [4] and are very resistant to proteolysis, providing much higher* in vivo* activity than the corresponding monomeric peptides [5–7].

We studied the use of oligobranched peptides containing the sequence of the human regulatory peptide neurotensin (NT4) as specific tumor targeting agents that can selectively and specifically deliver effector units for cell imaging or killing to tumor cells [6, 8]. We proved that NT4 can efficiently and selectively deliver functional units or liposomes [9] for cell imaging or therapy to different human cancer cells. Using NT4 conjugated to methotrexate or 5FdU, we obtained 60% and 50% reductions, respectively, in adenocarcinoma tumor growth in HT-29 xenografted nude mice [5, 6].

In the present study, NT4 was tested* in vitro* on HT-1376 and T24 bladder cancer (BC) cell lines (*in vitro *Phase I) and* ex vivo* on human BC samples from patients undergoing radical cystectomy or endoscopic transurethral resection of the bladder and the healthy tissue counterpart of the same patient (*ex vivo* Phase I) to evaluate its ability to recognize specific membrane receptors and to be internalized. Drug-conjugated NT4 and the corresponding free drug were compared* in vitro* (*in vitro* Phase II) to evaluate the capacity of NT4 to enhance the cytotoxic effect of the drug. An upcoming* ex vivo *Phase II on BC patients will be carried out in order to test tracers and chemotherapeutics conjugated with NT4.

## 2. Materials and Methods

### 2.1. Peptide Synthesis

Peptides were synthesized on an automated multiple synthesizer (MultiSynTech, Germany) by standard Fmoc chemistry. Protected L-amino acids and Tentagel-resin were purchased from Iris Biotech, Germany, DIPEA (N,N-diisopropylethylamine) from Merck and HBTU (O-benzotriazole-N,N,N′,N′-tetramethyl-uronium-hexafluoro-phosphate) from MultiSynTech.

Tetrabranched peptides were built using two consecutive Fmoc-Lys(Fmoc)-OH coupling steps. NT4-biotin was synthesized on Tentagel resin with Fmoc-Lys(biotin)-OH as first coupling step and Fmoc-PEG_12_-OH as second; Fmoc-Lys(Fmoc)-OH was then used to build the tetrameric core. Pyro-glu-O-pentachlorophenylester (Bachem, Switzerland) was used for the last coupling step, since pyro-glu is the N-terminal acid of the neurotensin sequence.

Peptides conjugated with chemotherapy drugs were synthesized using Fmoc-Lys(Dde)-OH as the first amino acid on Tentagel resin; then the second amino acid was Fmoc-*β*Ala-OH. Two coupling steps with Fmoc-Lys(Fmoc)-OH were used to build the core. Pyro-glu-O-pentachlorophenylester (Bachem, Switzerland) for NT(1–13) was the N-terminal acid. Once the sequence was completed, the Dde protective group was removed using 2% hydrazine in DMF (v/v) on resin and the free amino group was coupled with Fmoc-PEG_12_-OH. The Fmoc group was then removed to enable coupling with Boc-methotrexate or gemcitabine-ester. Gemcitabine-ester was prepared as already reported, with slight modification [6]. Unrelated analogues were synthesized similarly and the N-terminus was acetylated before the conjugation steps. Once the solid phase synthesis was completed, the peptides were cleaved from the resin, deprotected, and lyophilized.

HPLC purification was performed on a C18 Jupiter Phenomenex column. Water (A) containing 0.1% TFA and methanol (B) were used as eluents. Gradients of B in 30 min were run at flow rates of 0.8 mL/min and 4 mL/min for analytical and preparatory procedures, respectively. All compounds were also characterized on an Ettan MALDI-TOF mass spectrometer (Amersham Biosciences, Buckinghamshire, UK).

### 2.2. Bladder Cancer Cell Culture

T24 urinary bladder transitional cell carcinoma and HT-1376 urinary bladder carcinoma cells were obtained from Istituto Zooprofilattico Sperimentale (Brescia). T24 and HT-1376 cells were grown in their recommended media, McCoy's 5A and Eagle's minimum essential medium (MEM), respectively, supplemented with 10% fetal bovine serum, 200 *μ*g/mL glutamine, 100 *μ*g/mL streptomycin, and 60 *μ*g/mL penicillin in a humidified atmosphere with 5% CO_2_ at 37°C.

### 2.3. *In Vitro* Phase I: Peptide Binding and Internalization

The binding and internalization of tracing unit-conjugated NT4 was tested in HT-1376 and T24 cell lines. 3 × 10^4^ cells/well were seeded on 24-well plates, grown for 24 hours, blocked for 30 min at 37°C with 3% BSA in TBS, and then incubated with NT4 peptide (5 *μ*M in TBS-0.3% BSA). Experiments were performed with peptide conjugated to biotin and then incubated with 0.5 *μ*g/mL SA-Cy3. After 30 minutes of incubation at room temperature, the cells were washed with 0.3% BSA in TBS and grown in medium for 1, 2, or 4 h at 37°C to allow peptide internalization. Subsequently, the cells were fixed with TBS 4% formalin; then plasma membranes were stained with Lectin-FITC (0.5 *μ*g/mL in TBS-0.3% BSA) incubated for 15 min at room temperature and nuclei were stained with DAPI (0.5 *μ*g/mL in TBS-0.3% BSA). Each step was followed by three washes in TBS. Peptide binding and internalization were analysed by confocal laser microscope (Leica TCS SP5) with 550_*λ*ex_ and 570–590_*λ*em_, 380_*λ*ex_ and 450–470_*λ*em_, and 488_*λ*ex_ and 530–550_*λ*em_ for Cy3, DAPI, and FITC, respectively.

### 2.4. *In Vitro* Phase II: Cytotoxicity of Drug-Conjugated Peptides

T24 and HT-1376 cells were plated at a density of 5 × 10^3^ per well in 96-well microplates. Different concentrations of free or NT4-conjugated drugs, from 0.15 to 30 *μ*M, were added 24 h after plating. Cells incubated with methotrexate-conjugated peptide were grown without changing the medium for 6 days. Cells treated with gemcitabine-conjugated peptide were washed after 2 h incubation and then were left 6 days at 37°C with the same medium. Growth inhibition was assessed by 3-(4,5-dimethylthiazol-2-yl)-2,5-diphenyltetrazolium bromide (MTT). Cytotoxicity of drug-conjugated NT4 was compared with that of the corresponding free drugs and of an unrelated tetrabranched peptide, identically conjugated to the same drug.

### 2.5. *Ex Vivo* Phase I: Human Tissue Collection and Analysis

Samples of BC (*K*) were collected from patients undergoing either transurethral resection of bladder or radical cystectomy at Urology Clinic I, University Hospital of Florence. Samples of morphologically normal bladder mucosa (*H*) were collected from the same patient. The presence of tumor and healthy tissue was checked by standard staining procedures. Tissue samples from BC and healthy mucosa were stained with NT4-biotin, followed by streptavidin-FITC. Samples were embedded in Tissue-Tek and stored in liquid nitrogen. 10 *μ*m thick sections, obtained with a Leica CM1850 UV, were dried at 37°C for 30 min, fixed with 4% formalin for 15 min at room temperature, and incubated in glycine 0.1 M for 12 hours at 4°C. Blocking with FBS for 30 min at 37°C was followed by incubation for 30 min at room temperature with NT4-Biotin (1 *μ*g/mL) and SA-FITC (0.5 *μ*g/mL). Each step was followed by washing with TBS. Controls were performed using an unrelated biotin-conjugated tetrabranched peptide. Peptide binding was analysed by confocal laser microscope (Leica TCS-SP5) with 488_*λ*ex_ and 530–550_*λ*em_. All images were processed using ImageJ software (NIH). All patients were given a written informed consent module to read and sign before surgery, including detailed explanations of tissue sample collection and management of sensitive data. This study was carried out with approval of the Ethics Committee, Careggi, Florence (Protocol number 2009/0018748, 27.04.2009).

### 2.6. Statistical Analysis

The electronic data from image analysis was expressed as pixel distribution in the green color range of the RGB scale. Data was further analysed using Graph Pad Prism 5.03 software. Descriptive statistics (mean ± standard deviation and range) were used to summarize variables, while the nonparametric Wilcoxon rank test for paired samples was used to compare fluorescence signals in healthy and tumor tissue. For cytotoxicity determination, EC_50_ values were calculated by nonlinear regression analysis. Values from untreated controls gave 100% cell viability. The level of significance was set at *P* < 0.05 for two-sided testing.

## 3. Results

### 3.1. *In Vitro* Phase I: Peptide Binding and Internalization

**Figure 1:**
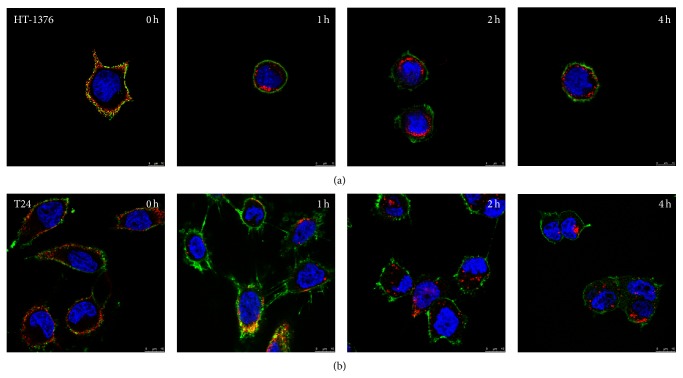
Binding and internalization (0 h, 1 h, 2 h, and 4 h) of NT4 (red) on HT-1376 human bladder cell carcinoma (a) and T24 urinary bladder transitional cell carcinoma (b). Nuclei are stained with DAPI (blue) and plasma membrane is stained with Lectin-FITC (green).

Binding and internalization of NT4 was tested in the HT-1376 cell line, which was chosen as representative of human bladder epithelial cell carcinoma, and in the T24 cell line, representative of transitional cell carcinoma. Cells were treated with 5 *μ*M NT4-biotin and then with streptavidin-Cy3 that provides a red signal. Peptide binding on membrane receptors was observed after 30 minutes of incubation at room temperature. Cells were then washed and incubated for a further 1, 2, or 4 h to follow peptide internalization. At time 0, NT4 red signal was localized on cell membranes. At the following incubation times, NT4 was clearly visualized intracellularly, both in HT-1376 and T24 cells (Figure 1).

### 3.2. *In Vitro* Phase II: Cytotoxicity of NT4 Conjugated to Chemotherapeutics

**Figure 2:**
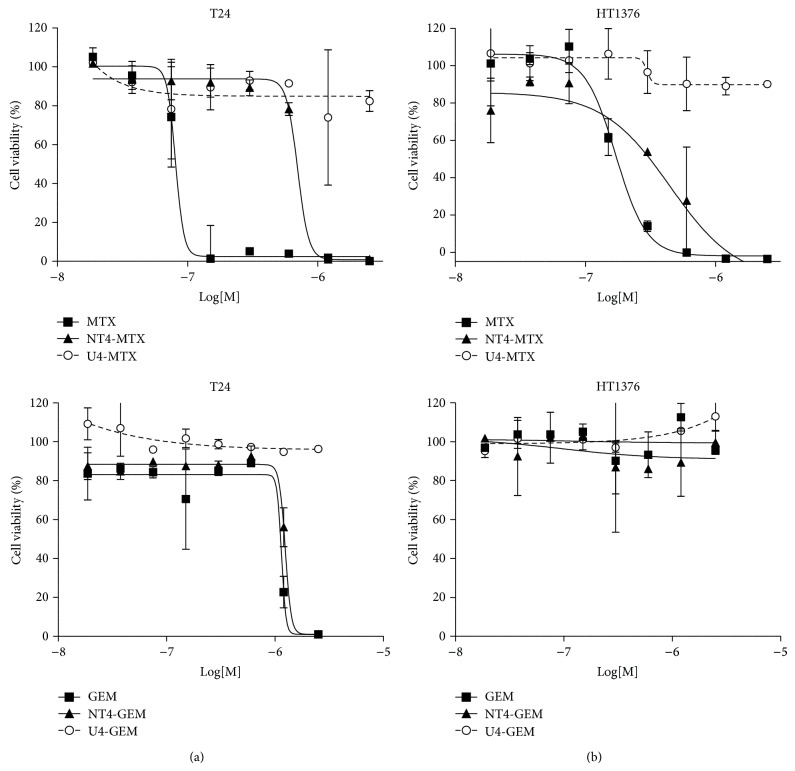
Cytotoxicity of NT4 peptide conjugated with methotrexate (MTX), or gemcitabine (GEM) in T24 (a) and HT-1376 (b) cell lines. Cytotoxicity of drug-conjugated NT4 (NT4-GEM or NT4-MTX) was compared with that of the corresponding free drugs (GEM or MTX) and an unrelated tetrabranched peptide (U4-GEM or U4-MTX), identically conjugated to the same drug.

Cytotoxicity of NT4 conjugated with methotrexate (MTX) or gemcitabine (GEM) was tested* in vitro* in HT-1376 and T24 bladder carcinoma cell lines. Drug-armed NT4 was tested for stability and ability to release the drug when incubated with cells. On the basis of the different links and bonds between NT4 peptides and the drugs, we classified drug-armed peptides as slow-releasing or fast-releasing adducts. Slow-releasing drug-armed NT4 released less than 10% of the conjugated drug in 24 hours, whereas fast-releasing adducts can release 50% of the conjugated drug in 2 hours of incubation [8]. MTX-conjugated NT4 was a slow-releasing adduct, whereas GEM-conjugated NT4 was fast-releasing.  Figure 2 shows the cytotoxicity of drug-conjugated NT4 compared with that of the corresponding free drugs and an unrelated tetrabranched peptide, identically conjugated to the same drug.

MTX was strongly active on HT-1376 and even more on T24. The conjugated peptide was less active than the free drug (EC_50_ 1.4 · 10^−7^ and 4.0 · 10^−7^ in HT-1376 and T24, resp.) but gained strong selectivity as shown by the curve obtained with the MTX-conjugated unrelated peptide U4-MTX. This result confirms that conjugation of MTX with NT4 turns the chemotherapeutic into an inactive prodrug, where MTX cannot exert its cytotoxic activity in the absence of peptide-dependent cell internalization [5, 6]. GEM and NT4-GEM were inactive on HT-1376 cells. NT4-GEM was as active as free GEM on T24.

### 3.3. *Ex Vivo* Phase I: Human Tissue Collection and Analysis

**Figure 3:**
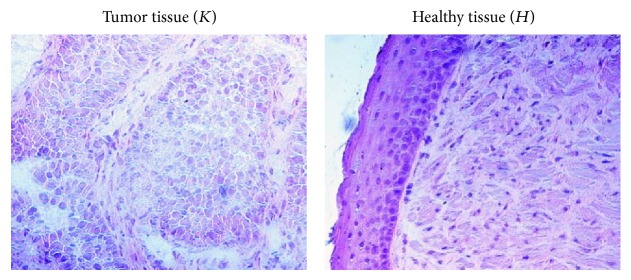
Human bladder specimens stained with hematoxylin and eosin (20x).

Samples from 16 patients who had undergone surgical resection of bladder cancer or endoscopic biopsy were obtained from the Urology Unit of Careggi Regional and University Hospital, Florence. Samples of morphologically normal tissue (healthy) from the same patients were collected from normal bladder mucosa. The presence of tumor and healthy tissue was checked by standard staining procedures (Figure 3).

**Figure 4:**
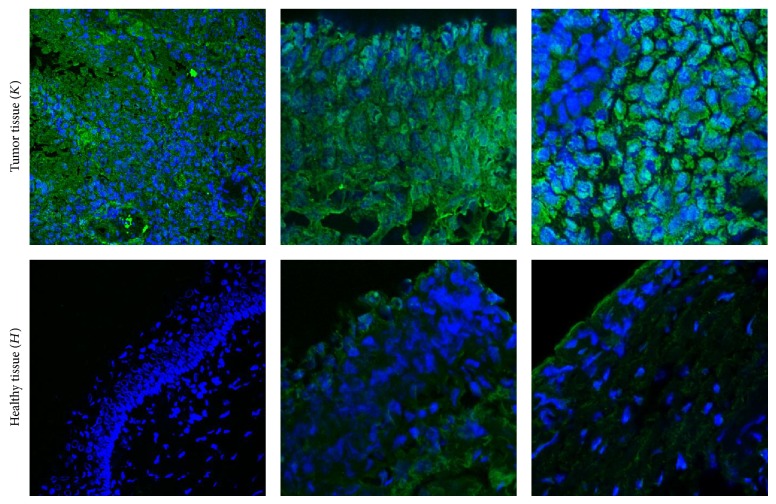
Confocal microscopy of human bladder cancer biopsies. Tumor tissue (*K*) and the corresponding healthy tissue (*H*) were stained with NT4 peptide conjugated with biotin, followed by streptavidin-FITC (green). Nuclei are stained with DAPI (blue).

Bladder cancer and healthy tissue samples were stained with NT4-biotin, followed by streptavidin-FITC (Figure 4).

A significant difference between tumor and healthy tissue signals was observed in all samples. RGB type images were collected and analysed to quantify fluorescent staining in each healthy and tumor tissue. RGB images were expressed as pixel distributions in the green color range using ImageJ software (NIH), translating the immunofluorescence signals into numbers representing the median value of green staining. Fourteen out of the 16 bladder cancers were diagnosed histologically as urothelial carcinoma (Table 1).

No correlation was found in the comparison between tumor staging (TNM in Table 1) and* K/H* value. This means that NT peptide can be used for early diagnosis and treatment of bladder cancer because the difference between tumor and healthy tissues in the early stages of the disease is substantial.

**Figure 5:**
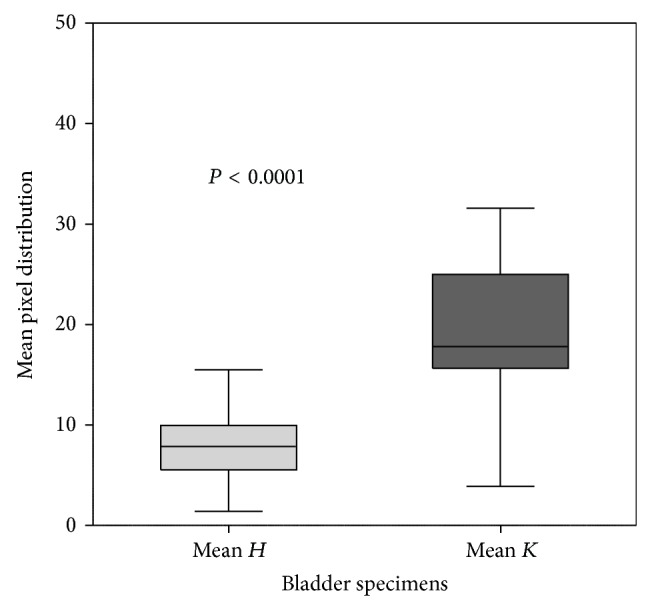
Comparison of mean fluorescence values in normal (mean* H*, light gray) and cancer (mean* K*, gray) tissue from human bladder specimens. The box represents the interquartile range (25–75th percentile) and the horizontal line in the box is the median value. Bottom and top bars of the whiskers indicate the minimum and maximum values, respectively.

Statistical analysis to evaluate any difference in peptide binding between healthy and tumor tissues showed a significant difference in fluorescent signal between the two groups (Figure 5). Indeed the signal from tumor samples was at least two-fold that, from the respective healthy tissue, is also visible from the* K/H* ratios shown in Table 1.

## 4. Discussion

Due to its high reoperation rate, bladder cancer has the highest lifetime treatment costs per patient of all cancers [10]. Bladder cancer is the fourth most common cancer among men and it is the ninth leading cause of death from cancer [11]. Recurrence and progression rate after endoscopic and intravesical treatments are 50–70% and 10–30%, respectively [12]. This leads to further endoscopic transurethral bladder resection or major surgery such as radical cystectomy.

Beside the intrinsic aggressiveness of bladder cancer, other critical aspects of this unsatisfactory outcome are related to suboptimal diagnosis of carcinoma* in situ* and flat tumors, which are not visualized in up to 39% of cases by common white light cystoscopy and to the limited efficacy of the current adjuvant treatments [13]. A recent study based on a long-term follow-up showed that 30% of patients treated with bacillus Calmette-Guérin had progression of the disease 2–6 weeks after transurethral resection [14, 15]. More effective adjuvant treatments are thus mandatory to reduce bladder cancer recurrence and progression.

The use of endogenous peptides for targeted personalized therapy has been envisaged as a promising approach in innovative selective cancer targeting [16]. Overexpression of tumor-associated antigens in several tumors can be capitalized by targeting these antigens with specific molecules, which might be selective for cancer cells.

Branched neurotensin sequences can confer the stability required for development of molecules with clinical purposes. Moreover, the three-lysine core offers additional chemical groups which can be used for conjugation of functional units outside the biologically active sequence. It has been demonstrated that NT4 branched neurotensin peptides are much more selective than native monomeric neurotensin in binding to different human cancer cells and tissues and that conjugation to different functional units does not affect its binding properties [5, 6].

In this study we tested T24 and HT-1376 cell lines, as representative of bladder cancer, for their binding and internalization of NT4 peptide. In both cases we observed complete peptide internalization within one hour. The same cell lines were also tested with NT4 peptide conjugated with chemotherapy drugs. When conjugated with NT4 peptide, methotrexate was strongly active against HT-1376 and even more so for T24.

As reported in previous studies [6, 8], although conjugation of a cytotoxic drug with NT4 may lead to a reduction in cytotoxicity* in vitro*, there may be a gain in selectivity and specificity which cannot be obtained with an unrelated branched peptide. This ultimately gives the NT4-conjugated drug higher* in vivo *activity than the free drug, as we already demonstrated by treating mice carrying xenografts of human colon adenocarcinoma cell lines with NT4-conjugated methotrexate and free methotrexate [5]. NT4-conjugated gemcitabine was as active against T24 cells as the free drug.

The* ex vivo* experiment on samples from patients undergoing surgery for bladder cancer showed that NT4 conjugated with a fluorescent probe discriminated between healthy and tumor tissues. Statistical analysis confirmed a substantial difference in fluorescence between healthy and tumor tissues with a heterogeneous response among patients. This finding substantiates evidence that the phenotype of a particular disease may vary between patients, so that treatments should be tailored on an individual basis. The results indicate that NT4 conjugated with fluorescent probes can be used in fluorescence cystoscopy, offering an improvement in the detection of flat neoplastic lesions, such as carcinoma* in situ*. The same molecule conjugated with chemotherapy drugs can be used in intravesical treatments to prevent disease recurrence and possible progression.

## 5. Conclusion

The modularity of the tetrabranched peptide could be a powerful feature because it allows the molecule to be armed with a therapeutic drug or with a probe for diagnosis [17]. The possibility of using the same molecule, firstly conjugated with fluorophores, to “label” cancer cells during endoscopy and then conjugated with chemotherapeutic drugs to treat tumors by bladder instillation, has important implications for a personalized approach to bladder cancer. The results indicate that NT4 peptides are a promising new selective tumor-targeting theranostic for human bladder cancer and may enable personalized oncology in which cancer diagnosis and therapy are obtained by means of the same molecule, with no modification in target binding but by simply “exchanging” the functional units.

## Figures and Tables

**Table 1:** Clinical features of bladder tumor samples.

Entry	Gender	Age	Type	*K*/*H*	TNM
1	M	72	Urothelial carcinoma	2,2	T2
2	M	82	Small cell carcinoma	7,5	T3b Nx Mx
3	M	66	Urothelial carcinoma	7,6	T3a N0 Mx
4	M	72	Urothelial carcinoma	2,8	T3a N0 Mx
5	M	55	Urothelial carcinoma	1,7	T2a N0 Mx
6	M	85	Urothelial carcinoma	2,3	Tx N1 Mx
7	F	80	Urothelial carcinoma	2,7	T4 Nx Mx
8	M	83	Urothelial carcinoma	1,1	T2b N0 Mx
9	M	83	Urothelial carcinoma	1,1	T2a N0 Mx
10	M	71	Urothelial carcinoma	2	T2
11	M	60	*In situ* carcinoma	4,4	Tis Nx Mx
12	M	81	Urothelial carcinoma	3,9	Ta Nx Mx
13	M	52	Urothelial carcinoma	2,2	Ta Nx Mx
14	M	72	Urothelial carcinoma	2,7	T1
15	M	73	Urothelial carcinoma	2,7	T2b N0 Mx
16	M	73	Urothelial carcinoma	1,2	T2b N0 Mx

*K*/*H* ratio was calculated on the median of *K* (tumor) and *H* (healthy) RGB values.
